# Computational and In Vitro Experimental Investigations Reveal Anti-Viral Activity of Licorice and Glycyrrhizin against Severe Acute Respiratory Syndrome Coronavirus 2

**DOI:** 10.3390/ph14121216

**Published:** 2021-11-24

**Authors:** Ahmed M. Tolah, Lamya M. Altayeb, Thamir A. Alandijany, Vivek Dhar Dwivedi, Sherif A. El-Kafrawy, Esam I. Azhar

**Affiliations:** 1 Special Infectious Agents Unit, King Fahd Medical Research Center, King Abdulaziz University, P.O. Box 128442, Jeddah 21362, Saudi Arabia; Altayeblmh@gmail.com (L.M.A.); talandijany@kau.edu.sa (T.A.A.); saelkfrawy@kau.edu.sa (S.A.E.-K.); 2 Department of Medical Laboratory Technology, Faculty of Applied Medical Science, King Abdulaziz University, P.O. Box 21911, Rabigh 344, Saudi Arabia; 3 Department of Medical Laboratory Technology, Faculty of Applied Medical Science, King Abdulaziz University, Jeddah 21589, Saudi Arabia; 4 Center for Bioinformatics, Computational and System Biology, Pathfinder Research and Training Foundation, Greater Noida 201308, India; Vivek_bioinformatics@yahoo.com

**Keywords:** COVID-19, SARS-CoV-2, licorice, glycyrrhizic acid, antiviral activity, Saudi Arabia

## Abstract

Without effective antivirals, the COVID-19 pandemic will likely continue to substantially affect public health. Medicinal plants and phytochemicals are attractive therapeutic options, particularly those targeting viral proteins essential for replication cycle. Herein, a total 179 phytochemicals of licorice (Glycyrrhiza glabra) were screened and scrutinized against the SARS-CoV-2 main protease (M^pro^) with considerable binding affinities in the range of −9.831 to −2.710 kcal/mol. The top 10 compounds with the best docking scores, licuraside, glucoliquiritin apioside, 7,3′-Dihydroxy-5′-methoxyisoflavone, licuroside, kanzonol R, neoisoliquiritin, licochalcone-A, formononetin, isomucronulatol, and licoricone, were redocked using AutoDock Vina, yielding −8.7 to −7.3 kcal/mol binding energy against Glycyrrhizin (−8.0 kcal/mol) as a reference ligand. Four compounds, licuraside, glucoliquiritin apioside, 7,3′-Dihydroxy-5′-methoxyisoflavone, and licuroside, with glycyrrhizin (reference ligand) were considered for the 100 ns MD simulation and post-simulation analysis which support the stability of docked bioactive compounds with viral protein. In vitro studies demonstrated robust anti-SARS-CoV-2 activity of licorice and glycyrrhizin under different treatment protocols (simulations treatment with viral infection, post-infection treatment, and pre-treatment), suggesting multiple mechanisms for action. Although both compounds inhibited SARS-CoV-2 replication, the half-maximal inhibitory concentration (IC50) of glycyrrhizin was substantially lower than licorice. This study supports proceeding with in vivo experimentation and clinical trials and highlights licorice and glycyrrhizin as potential therapeutics for COVID-19.

## 1. Introduction

The new Coronavirus Disease 2019 (COVID-19) has become a heath nuisance all over the world due to a lack of specific and potent medication [1,2,3]. Severe Acute Respiratory Syndrome Coronavirus 2 (SARS-CoV-2), the causative agent of the COVID-19 pandemic, can transmit from one infected person to another through aerosols [4]. SARS-CoV-2 infections have passed around 252 million confirmed cases and around five million deaths worldwide as of 12 November 2021, with no promising solution looming around to break the chain of the viral infection [5]. Researchers are trying to find out the solution for this problem using a drug repurposing approach. Remdesivir, ivermectin, hydroxychloroquine, and doxycycline in combination with other drugs are the most popular repurposed drugs which are being used to treat COVID-19 patients [6,7,8,9]. Further, researchers put tremendous effort to find new potential molecules which can independently block or interfere with viral replication in infected hosts [10].

Understanding the viral genome structure and replication cycle are key for identifying novel therapeutic potentials. The genomic constituent of SARS-CoV-2 is about 30 kb in size, which is translated into a polyprotein and further processed into non-structural, structural, and accessory proteins [11,12]. These proteins are responsible for the formation of new virus particles inside a host cell [12]. The non-structural proteins of SARS-CoV-2 have been recognized as potential drug-targets due to their significant role in viral genome processing and assembly [13]. The main protease (M^pro^), a non-structural protein of SARS-CoV-2, is a highly conserved and key target protein for antiviral drug discovery against COVID-19 due to its significant role in viral polyprotein processing [14]. Several crystallographic structures of SARS-CoV-2 M^pro^ have been solved and deposited in protein databank by different research groups [15,16,17,18]. Various natural compounds inhibiting SARS-CoV-2 M^pro^ have been reported in many research articles through in silico approaches, but their antiviral activity in vitro or in vivo were unreported.

Medicinal plants contain natural compounds with a wide range of diversity of chemical structures, thus there is a high probability of finding new lead molecules against various infections and diseases. Glycyrrhiza glabra (licorice) is one among those plants, which is well known for its potential antiviral activity against several DNA and RNA viruses (e.g., hepatitis viruses, herpes viruses, and pathogenic coronaviruses [19,20,21,22,23]). The antiviral role of licorice relies mainly on the inhibition of viral attachment on target cells either by binding to the viral glycoproteins or blocking the cellular receptors [21]. Evidence of licorice-induced antioxidative and anti-inflammatory activity during viral infections has also been demonstrated [21]. During the current COVID-19 era, glycyrrhizin (the active compound of licorice) has been used as a therapeutic option in China [24]. The anti-SARS-CoV-2 role of glycyrrhizin was linked to inhibitory activity against the viral spike (S) protein, which is key for viral uptake [25]. However, the antiviral role of Glycyrrhiza glabra (licorice) against other SARS-CoV-2 is not well characterized. Hence, this study aimed to identify the natural compounds in licorice with considerable potential to treat COVID-19 infection via targeting SARS-CoV-2 M^pro^. In this context, receptor-based drug discovery methods, including virtual screening, molecular docking, molecular dynamics (MD) simulation, and end point binding free energy approaches, were used to assess the therapeutic potential of the phytochemical compounds of licorice against SARS-CoV-2 by targeting its M^pro^ protein. The in vitro anti-SARS-CoV-2 activity of licorice was also evaluated.

## 2. Results and Discussion 

### 2.1. Virtual Screening and Re-Docking

Screening of natural compounds against a particular disease target may explore the possibility of new effective inhibitors with higher binding affinity and low toxicity. Virtual screening (VS) is computational technique, which is used for this application to save time and repetition of unnecessary experiments. In the present study, a total of 179 phytochemicals of licorice were screened against the SARS-CoV-2 M^pro^, and the result revealed the binding affinities of all the generated ligand poses in the range of −9.831 to −2.710 kcal/mol (Supplementary Table S1). The best poses of the top 10 compounds (licuraside, glucoliquiritin apioside, 7,3′-Dihydroxy-5′-methoxyisoflavone, licuroside, kanzonol R, neoisoliquiritin, licochalcone-A, formononetin, isomucronulatol, and licoricone) were selected for re-docking studies. Further, the best re-docked poses of the top 10 compounds were considered for molecular interaction analysis with the target protein. 

Various types of interactions, such as non-covalent interactions, electrostatic interactions, van der Waals interactions, salt bridges, hydrogen bonding, and metal interactions, are known to contribute a key role in the construction and stability of protein–ligand complexes. Remarkably, hydrogen bonding was reported to mediate ligand binding with the receptor and fundamentally contribute to the physiochemical properties of the molecules, which are essentially required for the drug development of lead compounds.

**Figure 1:**
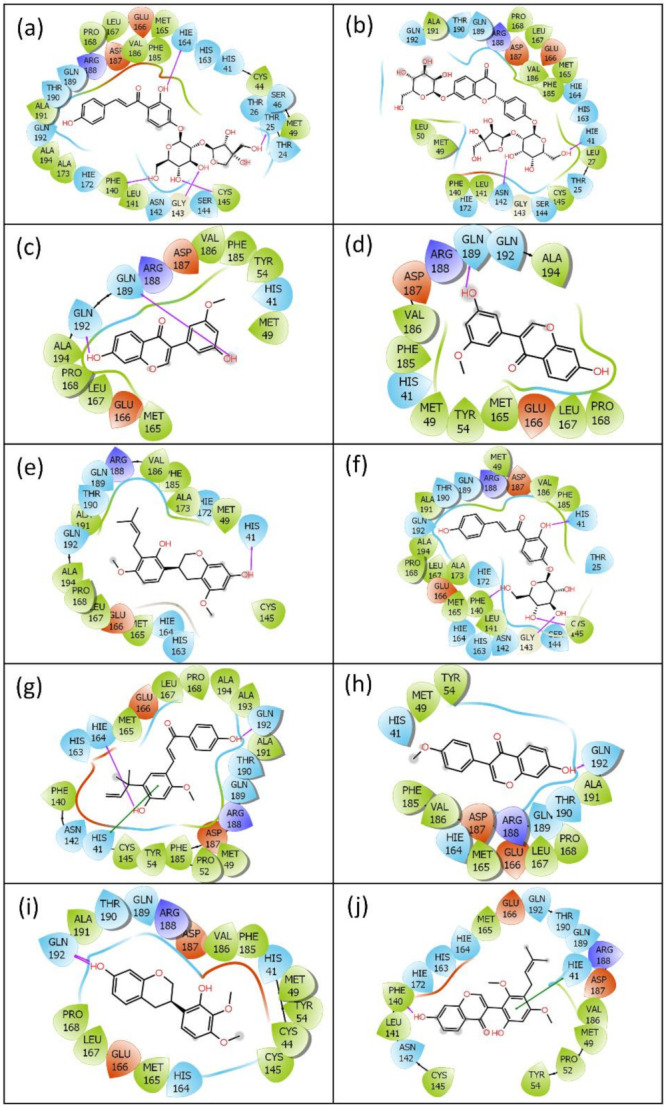
The 2D interaction poses for the docked poses of SARS-CoV-2 M^pro^ with selected bioactive compounds: (**a**) licuraside, (**b**) glucoliquiritin apioside, (**c**) 7,3′-Dihydroxy-5′-methoxyisoflavone, (**d**) licuroside, (**e**) kanzonol R, (**f**) neoisoliquiritin, (**g**) licochalcone-A, (**h**) formononetin, (**i**) isomucronulatol, and (**j**) licoricone. Herein, hydrogen bond formation (pink arrows), hydrophobic (green), polar (blue), red (negative), violet (positive), glycine (grey), and π-π stacking (green line), interactions are also depicted in the respective docked complexes.

Receptor residues and contact types involved in the molecular interaction with the top 10 compounds are given in Table 1. Interaction results revealed that the substrate binding residues of the receptor molecule were found to be involved in molecular contacts with the ligand molecules, as shown in Figure 1. The molecular interaction between SARS-CoV-2 M^pro^ and reference molecule (glycyrrhizin) is shown in Supplementary Figure S1 and interacting residues and contact type are given in Table 1. All the re-docked compounds were showing the molecular hydrogen bonding with most of the substrate binding residues of the target protein. Licuraside formed hydrogen bonds with one of the catalytic dyad residues (Cys145) and other important substrate binding residues. Glucoliquiritin apioside and kanzonol formed hydrogen bonds with another catalytic dyad residue (Hie41), while neoisoliquiritin formed hydrogen bonds with both the catalytic dyad residues and Gly143. No catalytic dyad residue was found to be involved in hydrogen bonding with the reference ligand. The top four compounds showed good binding affinity when compared to the reference molecule; hence, these four compounds were selected for further analysis using MD simulations and free binding energy calculations. 

### 2.2. Molecular Dynamics Simulation Analysis

**Figure 2:**
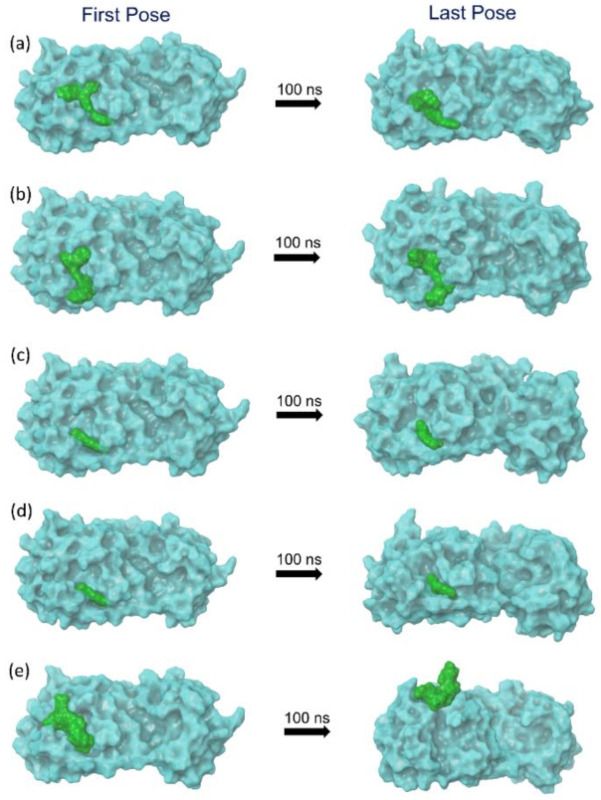
The 3D docked poses of SARS-CoV-2 M^pro-^bioactive compounds, namely natural products: (**a**) licuraside, (**b**) glucoliquiritin apioside, (**c**) 7,3′-Dihydroxy-5′-methoxyisoflavone, (**d**) licuroside, (**e**) glycyrrhizin, displaying the change in ligand conformation in the active pocket of SARS-CoV-2 M^pro^ during a 100 ns MD simulation interval.

**Figure 3:**
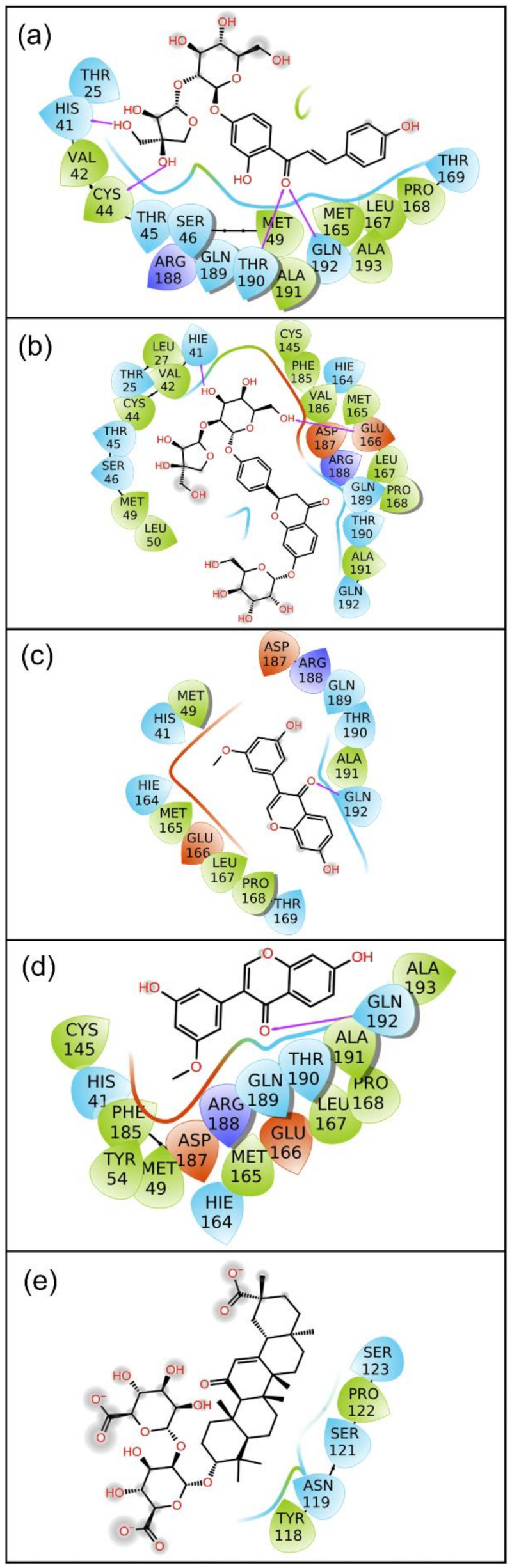
The 2D interaction diagram for the last poses of SARS-CoV-2 M^pro^ docked with bioactive compounds after MD simulations: (**a**) licuraside, (**b**) glucoliquiritin apioside, (**c**) 7,3′-Dihydroxy-5′-methoxyisoflavone, (**d**) licuroside, and (**e**) glycyrrhizin, extracted from 100 ns MD simulation. Herein, hydrogen bond formation (pink arrows), hydrophobic (green), polar (blue), red (negative), violet (positive), glycine (grey), and π-π stacking (green line) interactions are also shown in the respective docked complexes.

Molecular dynamics simulation is one of the integral parts of computational aided drug discovery approaches used to understand the binding stability of docked ligands in the active pocket of a receptor. In this study, the selected potential bioactive compound docked poses with SARS-CoV-2 M^pro^ were evaluated for their stability and intermolecular interaction formations by comparison to the reference complex via 100 ns MD simulation. Initially, the last poses from the 100 ns MD simulation trajectories of each complex were analyzed for the steadiness in the binding pocket of SARS-CoV-2 M^pro^ against their respective initial poses (Figure 2). Of note, all the selected bioactive compounds: licuraside, glucoliquiritin apioside, 7,3′-Dihydroxy-5′-methoxyisoflavone, and licuroside, showed substantial stability in the active pocket of viral protease by comparison to the reference complex, i.e., SARS-CoV-2 M^pro^-Glycyrrhizin complex, which showed displacement from the docked site in the viral protease (Figure 2). Moreover, each extracted last pose from the 100 ns MD trajectory were also studied for the formation of molecular contacts with the residues of the SARS-CoV-2 M^pro^. Interestingly, all the selected bioactive compounds exhibited considerable interaction with the active residues in the active pocket of viral protease against the reference complex. Herein, at least one hydrogen bond formation was noted, in addition to other intermolecular interactions, between the docked bioactive compounds and active residues in the binding pocket of SARS-CoV-2 M^pro^ (Figure 3; Table 2). However, no hydrogen bond or other substantial interactions were noted for the reference complex as its displacement from the active pocket during the 100 ns MD simulation (Figure 3; Table 2). Thus, selected bioactive compounds were marked as putative inhibitors of SARS-CoV-2 M^pro^; hence, respective MD trajectories were analyzed to obtain insights on docked complex stability via root mean square deviation (RMSD), root mean square fluctuation (RMSF), and total protein–ligand contact formation during 100 s MD simulation intervals. 

#### 2.2.1. RMSD and RMSF Analysis

**Figure 4:**
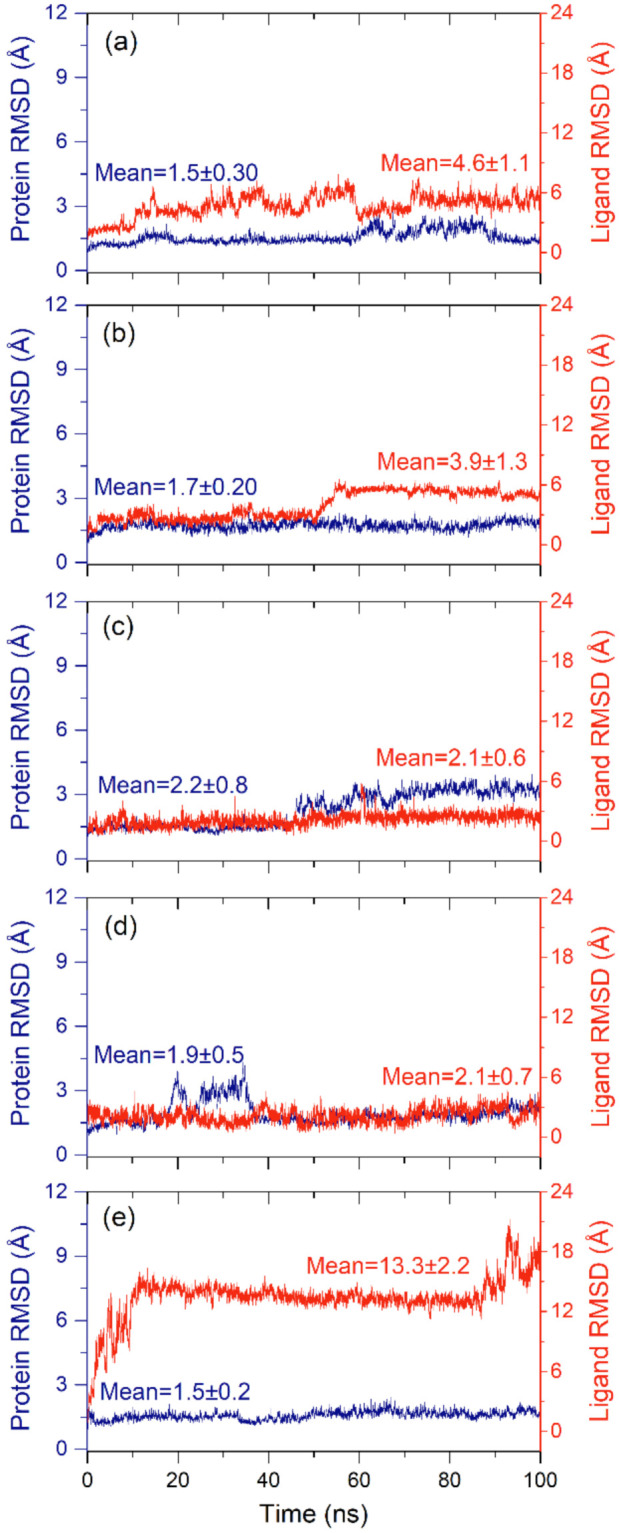
RMSD plots for the alpha carbon atoms (blue curves) of SARS-CoV-2 M^pro^ and protein fit ligand (red curves) were computed for the docked complexes of SARS-CoV-2 M^pro^ with selected compounds: (**a**) licuraside, (**b**) glucoliquiritin apioside, (**c**) 7,3′-Dihydroxy-5′-methoxyisoflavone, (**d**) licuroside, and (**e**) glycyrrhizin, obtained from the 100 ns MD simulation trajectory.

**Figure 5:**
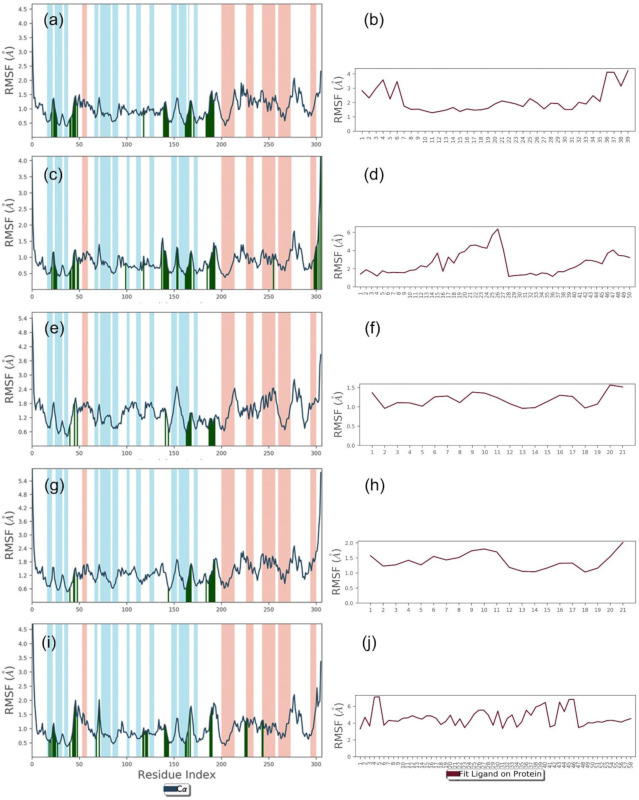
RMSF values plotted for alpha carbon atoms of viral protease and bioactive compounds fit on protein structure in the docked complexes of SARS-CoV-2 M^pro^ with docked with natural products with selected compounds: (**a**,**b**) licuraside, (**c**,**d**) glucoliquiritin apioside, (**e**,**f**) 7,3′-Dihydroxy-5′-methoxyisoflavone, (**g**,**h**) licuroside, and (**i**,**j**) glycyrrhizin, obtained from the 100 ns MD simulation trajectory, extracted from the 100 ns MD simulation interval.

To collect the average displacement in the protein structure and bioactive compound in each docked complex during the 100 ns MD simulation interval, all the frames of the MD simulation trajectory were aligned to the initial frame and RMSD value for protein structure (Cα) and protein fit ligand were calculated (Figure 4). Of note, all the protein structures in docked complexes with selected bioactive compounds showed acceptable RMSD values (<2.2 Å) by comparison to the reference docked complex (<1.5 Å) until the end of the 100 ns MD simulation (Figure 4). These results suggested the stability of the viral protein in the docked complexes with selected bioactive compounds during MD simulation interval. Likewise, computed RMSD values for the protein-fit ligand also showed acceptable deviations (<4.6 Å) during the simulation interval by comparison to the reference ligand (<13.2 Å) until the end of the 100 ns simulation interval. Of note, 7,3′-Dihydroxy-5′-methoxyisoflavone (<2.1 ± 0.6 Å) and licuroside (<2.1 ± 0.6 Å) showed substantial stability and gained equilibrium state within 10 ns against other selected bioactive compounds, i.e., licuraside (<4.6 ± 1.1 Å) and glucoliquiritin apioside (<3.9 ± 1.3 Å), and reference complex (<13.3 ± 2.2 Å), during the 100 ns MD simulation (Figure 4). Furthermore, calculated RMSD values for the docked protein with bioactive compounds were supported by acceptable RMSF values (<1.2 Å), except in the residues interacting with the docked ligands (<2 Å) and N- and C-terminal of the protein structure (<5.5 Å) (Figure 5). Likewise, the docked bioactive compound as the fit ligand on protein structure was also noted for acceptable RMSF values (<3 Å) against the reference ligand (<4 Å), except during higher fluctuations (<7 Å) in the atoms contributing intermolecular contact formation with the active residues of the protein during the MD simulation interval (Figure 5). Collectively, all the selected bioactive compounds of licuraside, glucoliquiritin apioside, 7,3′-Dihydroxy-5′-methoxyisoflavone, and licuroside, were marked for substantial stability in the active pocket of viral protease against the reference compound, i.e., glycyrrhizin.

#### 2.2.2. Protein–Ligand Contact Mapping

**Figure 6:**
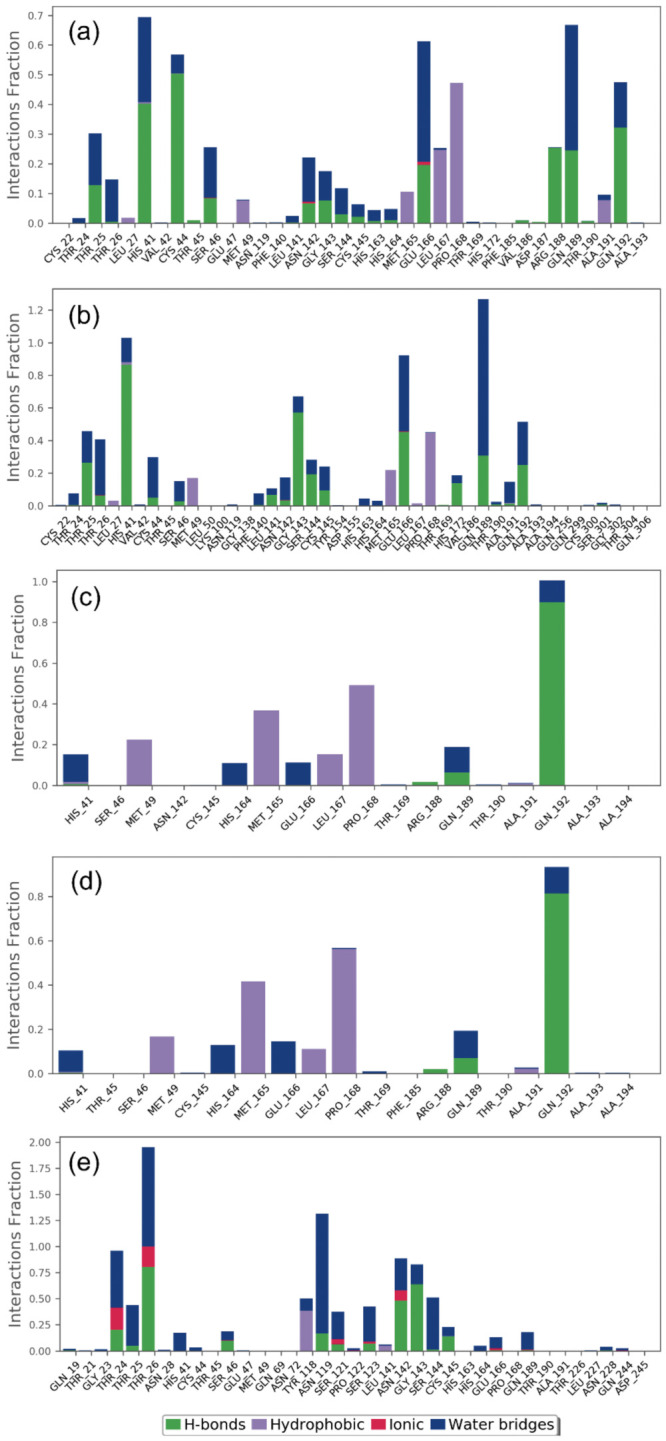
Protein–ligand interaction contacts profiling extracted during a 100 ns MD simulation for SARS-CoV-2 M^pro^ docked with selected compounds: (**a**) licuraside, (**b**) glucoliquiritin apioside, (**c**) 7,3′-Dihydroxy-5′-methoxyisoflavone, (**d**) licuroside, and (**e**) glycyrrhizin.

In drug design, hydrogen bonding (backbone acceptor; backbone donor; side-chain acceptor; side-chain donor) has been reported as an essential factor to decipher the metabolism, adsorption, and specificity of the drug candidate. Besides this, the substantial role of non-covalent interactions, including hydrophobic interactions, π-Cation; π-π; polar or ionic interactions, and the formation of a water-bridge hydrogen bond, were also elucidated in the establishment of docked protein–ligand poses during the MD simulation interval. Therefore, intermolecular interactions were extracted for SARS-CoV-2 M^pro^ with docked with bioactive compounds, namely licuraside, glucoliquiritin apioside, 7,3′-Dihydroxy-5′-methoxyisoflavone, and licuroside, against the reference compound glycyrrhizin, were mapped from the respective MD simulation trajectory under the default parameters of the Desmond module (Figure 6). 

Interestingly, all the docked bioactive compounds and the reference compound in the active pocket of SARS-CoV-2 M^pro^ showed substantial molecular contact formation with active residues, i.e., His41, Cys145, and Gln192, required for catalysis and substrate binding in the viral protease (Figure 6). Of note, these interacting residues were also noted in the respective docked complexes (Table 1, Figure 1), indicating the stability of docked ligands in the active pocket during the simulation interval as also predicted from the last pose analysis (Figure 2 and Figure 3). Moreover, all the docked compounds exhibited considerable molecular contact formation during the 100 ns MD simulation interval, including hydrogen bonding, water bridging, hydrophobic, and ionic interactions, with the essential residues in the binding pocket of SARS-CoV-2 M^pro^ (Figure 6) Convincingly, analysis of the protein–ligand contact mapping advised the significant residence of selected bioactive compounds in the catalytic pocket of SARS-CoV-2 M^pro^ by comparison to the reference compound. Hence, the bioactive compounds from Glycyrrhiza glabra can be marked in the descending order. i.e., (a) licuraside, (b) glucoliquiritin apioside, (c) 7,3′-Dihydroxy-5′-methoxyisoflavone, and (d) licuroside, as potent SARS-CoV-2 M^pro^ inhibitors based on the number of molecular contacts formation during the 100 ns MD simulation interval. 

### 2.3. Binding Free Energy Analysis

**Figure 7:**
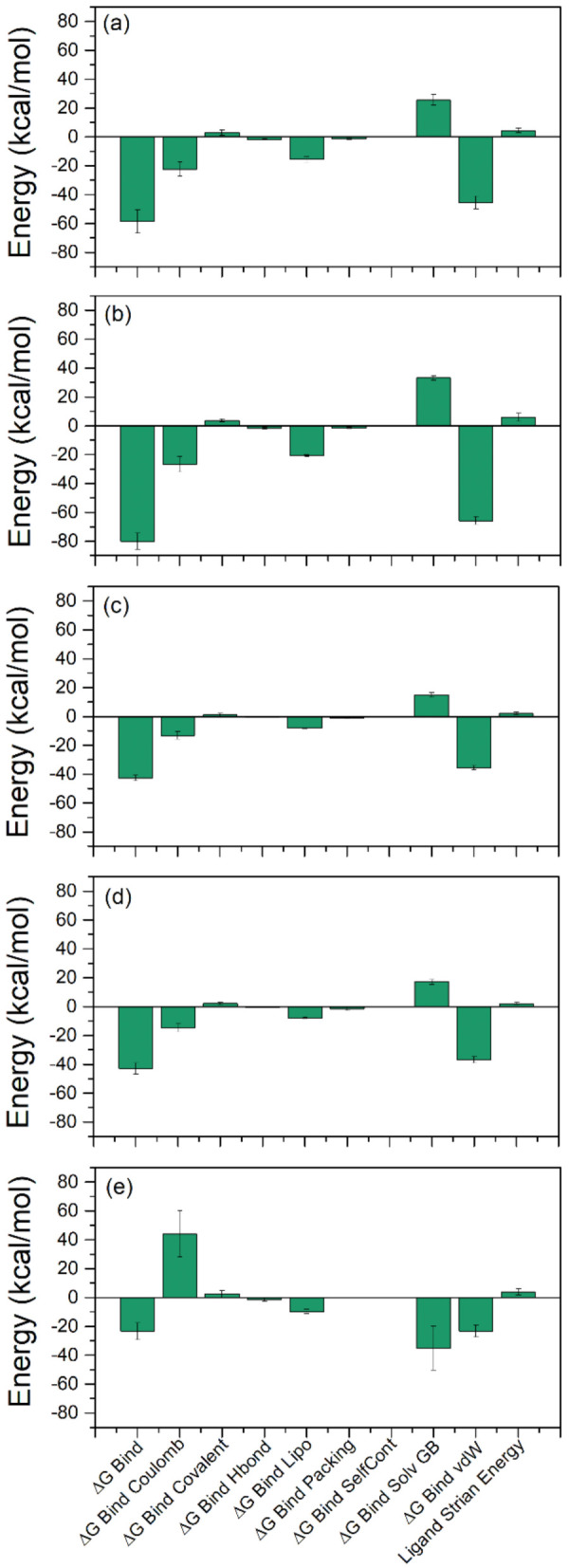
Binding free energy calculated for the snap shots for SARS-CoV-2 M^pro^ complexes with potential bioactive compounds, i.e., (**a**) licuraside, (**b**) glucoliquiritin apioside, (**c**) 7,3′-Dihydroxy-5′-methoxyisoflavone, (**d**) licuroside, and (**e**) glycyrrhizin, from licorice.

Computational calculations, such as MD-based MMGBSA methods to calculate the binding free energy for the protein–ligand complex, have been reported as rapid and cost-effective approaches to identifying potent inhibitors. Hence, binding free energy of docked bioactive compounds in the selective pocket of SARS-CoV-2 M^pro^ (ΔG_Bind_) were determined using an MM/GBSA approach [26], implemented in the prime MM/GBSA module of Schrödinger Suite. Herein, average binding free energy was computed using the extracted protein–ligand poses from the last 10 ns simulation interval of 100 ns MD simulation trajectory. Interestingly, all the selected bioactive compounds displayed considerable binding free energy values, where a maximum and minimum of binding free energy values were markedly noted for SARS-CoV-2 M^pro^-Glucoliquiritin −80.0 ± 5.60 kcal/mol) and SARS-CoV-2 M^pro^-7,3′-Dihydroxy-5′-methoxyisoflavone (−42.73 ± 1.94 kcal/mol) complexes, respectively. Moreover, the contribution of individual energy components to the total binding free energy, i.e., ΔG_Bind Coulomb_, ΔG_Bind Covalent_, ΔG_Bind Hbond_, ΔG_Bind Lipo_, ΔG_Bind Packing_, ΔGB_ind SelfCont_, ΔG_Bind Solv GB_, and ΔG_Bind vdW_, were also computed in the MM/GBSA method (Table 3, Figure 7). Remarkably, ΔG_Bind Coulomb_ and ΔG_Bind vdW_ were noted for major contributions in favorable binding free energy for the docked compounds with the viral protease. Furthermore, ΔG_Bind Covalent_ and ΔG_Bind Solv GB_ were observed for involvement in unfavorable energy, and hence decreased the net binding free energy values for each docked protein–ligand complex. The observed results were similar to the recent reported MMGBSA results for SARS-CoV-2 M^pro^ docked complexes with FDA drugs, where ΔG_Bind Coulomb_ and ΔG_Bind vdW_ indicated the highest contribution in the stability of respective docked complexes [3]. Hence, based on collective data analysis, the selected bioactive compounds were inferred for significant stability in the active pocket of SARS-CoV-2 M^pro^ as projected from molecular docking and MD simulation analysis.

### 2.4. Assessment of Licorice and Glycyrrhizin Anti-SARS-CoV-2 Activity In Vitro

**Figure 8:**
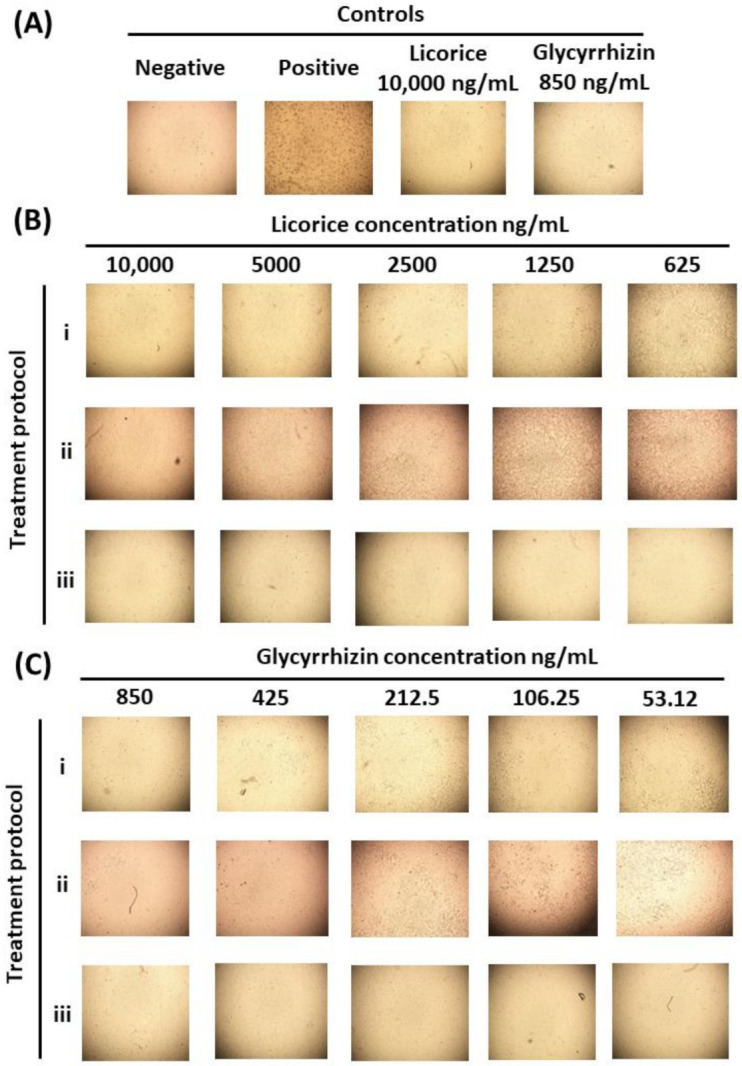
In vitro assessment of licorice and glycyrrhizin antiviral activity against SARS-CoV-2. Representative images of (**A**) controls: negative (uninfected cells), positive (infected cells), cytotoxicity controls (licorice- and glycyrrhizin-treated uninfected cells). (**B**) Antiviral effects of licorice at a range of concentrations on SARS-CoV-2 infected cells, and (**C**) antiviral effects of licorice at a range of concentrations on SARS-CoV-2 infected cells. Details about treatment protocols (**A**–**C**) are found in Section 3.5.4. (the effect of licorice and glycyrrhizin on viral induced cytopathic effect (CPE) and viral plaque forming efficiency (PFE)).

**Figure 9:**
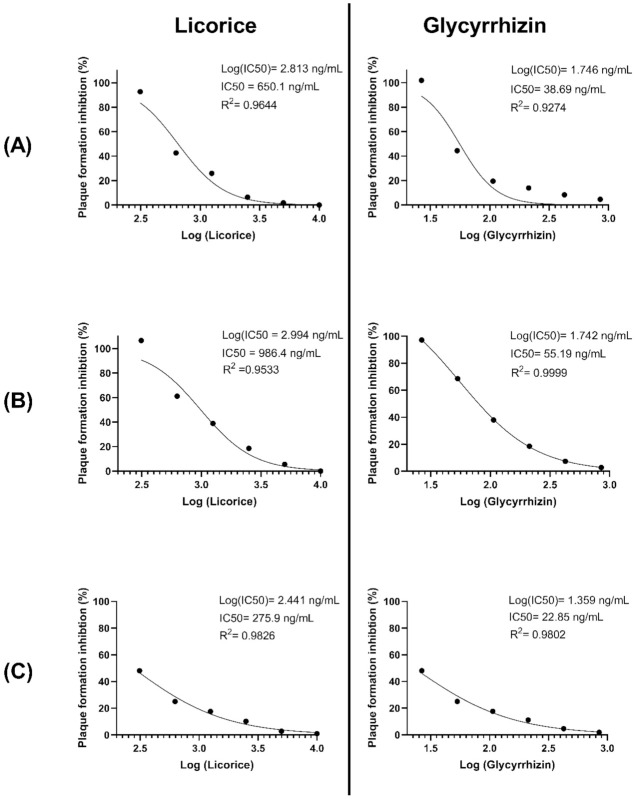
Calculation of the half maximal inhibitory concentration (IC50) of licorice and glycyrrhizin on SARS-CoV-2 plaque forming efficiency. The IC50, log (IC50), and R squared (R2) for each compound under each treatment protocol are shown. Details about treatment protocols (**A**–**C**) are found in Section 3.5.4. (the effect of licorice and glycyrrhizin on viral induced cytopathic effect (CPE) and viral plaque forming efficiency (PFE)).

Although computational drug design approaches are valuable for the screening of potential antivirals, laboratory experimentation remains important to validate the findings. Herein, we have investigated the effects of licorice and glycyrrhizin on the viral-induced CPE. Moreover, three different treatment protocols have been utilized: (A) simultaneous addition of virus and compound to the cells, (B) treatment of cells post-viral entry, and (C) pretreatment of infected cells prior to infection. The range of concentrations utilized was as follows: licorice between 312.5 and 100 ng/mL and glycyrrhizin between 26.5 ng and 850 ng/mL. These concentrations were chosen based on data obtained from a cell cytotoxicity assay (data not shown). At these non-toxic concentrations and under all three treatment protocols, both licorice and glycyrrhizin substantially inhibited the virus-induced CPE and PFE. In fact, there was a complete lack of CPE at the highest treatment concentrations. Moreover, these inhibitory effects appear to occur in dose-dependent manners (Figure 8). Our in silico work suggested an inhibitory effect of licorice on the viral M^pro^ while others identified an effect on the viral S protein. The inhibitory effects on viral replication under the three treatment conditions propose more than a single mechanism of actions. This was supported by the IC50 of each compound under each treatment condition (Figure 9), with pre-treatment protocols having the lowest IC50 values. Moreover, the IC50 of glycyrrhizin was substantially lower than licorice. This was expected as glycyrrhizin represents the active compound.

Collectively, our computational and in vitro experimental investigations revealed anti-viral activity of licorice and glycyrrhizin against SARS-CoV-2 via the targeting of Mpro. This study, in addition to previous work that characterized licorice-induced anti-S activity, poses another forward step to understanding the anti-SARS-CoV-2 role of licorice. Detailed investigations of the antiviral effects (e.g., the effect on viral gene expression, protein synthesis, and direct interaction with viral proteins) are still required to obtain a comprehensive picture about these mechanisms of action. In vivo studies and clinical trials are also urged to enhance the potential of utilizing licorice and glycyrrhizin as therapeutic options for COVID-19.

## 3. Materials and Methods

### 3.1. Receptor and Ligand Structure Data Collection

A 3D high-resolution structure of the SARS-CoV M^pro^ in combination with Narlaprevir was searched and fetched from a PDB database with PDB ID: 7JY [27]. The structure was solved at 1.79 Å resolution using X-ray crystallography. In the target protein, the substrate binding residues of the protein are present in combination with Narlaprevir. PubChem database was used to retrieve the 3D structures of 179 licorice phytochemicals [28].

### 3.2. Structure Preparation and Virtual Screening

The 3D structures of SARS-CoV-2 M^pro^ were prepared using Dock Prep tool inbuilt in Chimera [29], and the prepared file was saved in pdb format. PyRx 0.8 was used for the preparation of ligands and the virtual screening (VS) experiment [30,31]. Briefly, ligands in sdf files of the phytomolecules were uploaded into the Open Babel program option of PyRx 0.8 software [30,31] and minimized using all option at default settings, and then optimized ligands were converted into pdbqt format. The native ligand binding region was selected to generate Grid box on the protein structure for the VS experiment using the AutoDock Vina tool in PyRx 0.8 [32]. After completion of the VS, the best poses of the top 10 compounds were selected based on their docking score.

### 3.3. Re-Docking and Interaction Analysis

Re-docking of the top 10 screened compounds was carried out in a Chimera-AutoDock Vina plugin [32,33] setup to confirm the binding residues of the M^pro^ with respective phytomolecules. Re-docking was performed with default settings at the pre-defined reference ligand binding site. The grid (30 Å × 30 Å × 30 Å) was set up along the three (X, Y, and Z) axes, covering the entire crucial residues of the target protein to provide enough space for the ligand binding. The best poses of re-docked compounds were selected for further molecular contact analysis in free academic Maestro (Schrödinger Release 2020-1: Maestro, Schrödinger, LLC, New York, NY, USA, 2020). Herein, non-covalent contacts were calculated to generate both 3D and 2D interaction images by selecting the residues around ligands at a 4 Å radius under default parameters [34]. Similar molecular docking parameters were also employed for the glycyrrhizin, a previously reported SARS-CoV-2 Mpro from licorice [35], for comparative examination with the selected phytochemicals.

### 3.4. Molecular Dynamics Simulation and Free Binding Energy Calculations

A 100 nanosecond molecular dynamics (MD) simulation of the top 4 compounds and reference ligand (glycyrrhizin) in a complex with a receptor molecule was performed using the free academic Desmond program (Maestro-Desmond Interoperability Tools, Schrödinger, New York, NY, 2018) [36]. For the system setup of each protein–ligand complex, a TIP4P solvent model was selected under an orthorhombic grid box (10 Å × 10 Å × 10 Å buffer) and minimized. Later, the salt and ion placements were excluded at 20 Å from the ligand. For the neutralization of the complete system, counter ions were added. System setup was performed in system builder tool of the Desmond program. After the completion of system building, the entire system for each complex was minimized using a Desmond minimization tool. Later, the MD simulations for each minimized system were run at 300 K temperature, and 20 ps NPT reassembly at 1 atm pressure. A 50 ps recording interval was set to generate 2000 frames. A 1000 ps relaxation time for the thermostat method and 2000 ps for the barostat method were set for system relaxation. After MD simulation, trajectories of each system were analyzed using a simulation interaction diagram tool from Desmond [34,36]. The MM/GBSA tool of the prime module of Schrodinger Suite (Schrödinger Release 2020-4: Prime, Schrödinger, LLC, New York, NY, USA, 2020) was used to calculate the net free binding energy of simulated last poses of each protein–ligand complex [2].

### 3.5. In-Vitro Assessment of Licorice and Glycyrrhizin Antiviral Activity against SARS-CoV-2

#### 3.5.1. Cell Line

African green monkey kidney cells Vero E6 (ATCC^®^ CRL-1586™) grown and maintained at 37 °C in 5% CO_2_ were used for in vitro work, including viral propagation and titration, and assessment of Licorice and Glycyrrhizin antiviral activity. 

#### 3.5.2. Virus and Compounds

A human SARS-CoV-2 clinical patient isolate (SARS-CoV2/human/SAU/85791C/2020, gene bank accession number: MT630432) was propagated and titrated as previously described [37]. All experiments were conducted at the Special Infectious Agents Unit (SIAU) Biosafety level 3 facility at King Fahd Medical Research Center (KFMRC), King Abdulaziz University (KAU), Jeddah, Saudi Arabia.

Licorice and glycyrrhizin were purchased from a local and online herbal store (INDOFINE Chemical Company, Inc. Lot no. # 0406216 Hillsboro, NJ, USA), respectively. The compound stocks were freshly prepared on the day of experiments in Dulbecco’s Modified Eagle Medium (DMEM) and purified using 0.22 µM filters prior to use.

#### 3.5.3. Cell Toxicity Assay

Cells were seeded in a 96-well tissue culture plate and incubated for 12 h prior to the addition of compounds. At 72 h post-treatment, 0.4% neutral red (Sigma, St. Louis, MO, USA) was added and incubated for 4 h at 37 °C in a 5% CO_2_. Then, the neutral red solution was discarded, and cells were fixed with 100 µL per well of 5% formaldehyde for 5 min at room temperature. Wells were washed with a sterile Dulbecco phosphate buffered saline (DPBS) solution prior to the addition of 100 μL lysis solution containing 50% ethanol (Sigma, St. Louis, MO, USA) and 0.01% acetic acid (Sigma, St. Louis, MO, USA). Plates were incubated for 10 minutes at room temperature on a shaker and then read by using an Elx 808 bioelisa reader (Biokit, Barcelona, Spain), the optical density was read at 540 nm (OD540).

#### 3.5.4. The Effect of Licorice and Glycyrrhizin on Viral Induced Cytopathic Effect (CPE) and Viral Plaque Forming Efficiency (PFE))

Cells were seeded and incubated for 12 h prior to proceeding with one of the following treatment protocols:

The compounds at a range of concentrations (from 312.5 to 10,000 ng/mL for licorice and from 26 to 850 ng/mL for glycyrrhizin) with multiplicity of infection (MOI) of 0.1 of SARS-CoV-2 were simultaneously added to the cells. Following an hour of viral adsorption at 37 °C, the cells were overlaid with media containing the compounds at the range of concentrations indicated for 72 h.

Cell were infected with an MOI of 0.1 of SARS-CoV-2. Following an hour of viral adsorption at 37 °C, the cells were overlaid with media containing the compounds at a range of concentrations (described above in (A)) for 72 h.

Cells were pretreated with the compounds at a range of concentrations (described above in A)) for an hour at 37 °C. Then, treated cells were infected with MOI of 0.1 of SARS-CoV-2 and overlaid with media containing compounds for 72 h.

Negative (uninfected cells) and positive controls (SARS-CoV-2 infected cells in the absence of treatment) were included in all experiments. The cells were checked for CPE under microscope on daily bases and images were obtained using a Nicon ECLIPSE Ti camera (Nicon DS-Fi1, NIS-Elements AR 3.2 software). For viral PFE, the same protocols were utilized. However, 0.8% agarose was added to the overlay in order to enable plaque formation. The numbers of plaques were counted, percentage of inhibition relative to positive control was determined, and the half-maximal inhibitory concentration (IC50) was calculated.

## 4. Conclusions

The role of SARS-CoV-2 main protease (M^pro^) in the proteolytic processing of the polyprotein inside a host cell makes it an attractive drug target for anti-SARS-CoV-2 drug development. Further, a broad range of phytochemicals as natural bioactive molecules in licorice (Glycyrrhiza glabra) have been reported with therapeutic benefits. Hence, in this study, the four selected bioflavonoids of licuraside, glucoliquiritin apioside, 7,3′-Dihydroxy-5′-methoxyisoflavone, and licuroside, out of the top 10 re-docked complexes obtained after screening of 179 molecules were subjected for molecular dynamics simulations and end-point binding free energy calculations. Re-docking analysis of the molecules showed that selected compounds formed strong molecular contacts with important residues of the SARS-CoV-2 M^pro^ active site. Molecular dynamics simulations and free binding energy calculations of four selected protein ligand complexes revealed the stability of bioactive compounds into the active site of the target molecule. These results supported licuraside, glucoliquiritin apioside, 7,3′-Dihydroxy-5′-methoxyisoflavone, and licuroside as potential inhibitors of SARS-CoV-2 M^pro^. Importantly, in vitro investigations demonstrated anti-SARS-CoV-2 activity of licorice (crude) and Glycyrrhiza. In vivo experimentation and clinical trials are needed to consider these compounds as therapeutic potentials for COVID-19 patients. Further, these selected compounds could be considered for the generation of lead inhibitors against SARS-CoV-2 using structure–activity relationships (SAR), which is widely used in drug discovery pipelines to predict biological activity from the existing molecular structures of ligands or inhibitors.

## Figures and Tables

**Table 1:** Intermolecular interactions of selected docked compounds and reference ligand in the catalytic pocket of SARS-CoV-2 M^pro^.

S. No.	Compound Name	Docking Score	H-Bond	π–π Stacking	Hydrophobic	Polar	Negative	Positive	Glycine
1	Licuraside	−8.7	Hie164, Phe140, Cys145, Gly143, Thr25	--	Cys145, Leu141, Phe140, Ala173, Ala194, Ala191, Pro168, Leu167, Val186, Phe185, Cys44, Met49, Met165	Thr24, Thr25, Thr26, Ser46, His41, His163, Hie164, Gln189, Thr190, Gln192, Hie172, Asn142, Ser144	Asp187, Glu166	Arg188	Gly143
2	Glucoliquiritin apioside	−8.8	Asn142, Hie41	--	Leu50, Met49, Phe140, Leu141, Cys145, Leu27, Met165, Phe185, Val186, Leu167, Pro168, Ala191	Gln192, Thr190, Gln189, Hie164, His163, Hie41, Thr25, Ser144, Asn142, Hie172	Asp187, Glu166	Arg188	Gly143
3	7,3′-Dihydroxy-5′-methoxyisoflavone	−8.1	Gln189, Gln192	--	Met49, Tyr54, Phe185, Val186, Ala194, Pro168, Leu167, Met165	His41, Gln189, Gln192	Asp187, Glu166	Arg188	--
4	Licuroside	−8.1	Gln189	--	Ala194, Val186, Phe185, Met49, Tyr54, Met165, Leu167, Pro168	Gln192, Gln189, His41	Asp187, Glu166	Arg188	--
5	Kanzonol R	−7.5	His41	--	Met165, Leu167,Pro168, Ala194, Ala191, Val186, Phe185, Ala173, Met49, Cys145	His41, Hie172, Gln189, Thr190, Gln192, Hie164, His163	Glu166	Arg188	--
6	Neoisoliquiritin	−8.7	His41, Cys145, Gly143	--	Phe185, Val186, Met49, Ala191, Ala194, Pro168, Leu167, Ala173, Met165, Phe140, Leu141, Cys145	Thr25, His41, Gln189, Thr190, Gln192, Hie172, Hie164, His163, Asn142, Ser144	Asp187, Glu166	Arg188	Gly143
7	Licochalcone-A	−8.0	Hie164, Gln192	His41	Met165, Leu167, Pro168, Ala194, Ala193, Ala191, Met49, Pro52, Phe185, Tyr54, Cys145, Phe140	His163, Hie164, Gln192, Thr190, Gln189, His41, Asn142	Asp187, Glu166	Arg188	--
8	Formononetin	−7.3	Gln192	--	Tyr54, Met49, Ala191, Pro168, Leu167, Met165, Val186, Phe185	His41, Gln192, Thr190, Gln189, Hie164	Asp187, Glu166	Arg188	--
9	Isomucronulatol	−8.0	Gln192	--	Ala191, Val186, Phe185, Met49, Tyr54, Cys145, Met165, Leu167, Pro168	Gln192, Thr190, Gln189, His41, His164	Asp187, Glu166	Arg188	--
10	Licoricone	−7.4	Phe140	Hie41	Cys145, Leu141, Phe140, Met165, Val186, Met49, Pro52, Tyr54	Asn142, Hie172, His163, Hie164, Gln192, Thr190, Gln189, Hie41	Asp187, Glu166	Arg188	--
11	Reference complex	−8.0	Thr25, Thr26, Asn142, Gly143	--	Leu27, Cys44, Met49, Leu167, Pro168, Ala191	Thr24, Thr25, Thr26, His41, Thr45, Ser46, Asn142, Gln189	Glu166	--	Gly143

**Table 2:** Analysis of molecular contacts in the last poses of each protein-ligand complex after MD simulation.

S. No.	Drug	H-Bond	Pi–Pi Stacking	Hydrophobic	Polar	Negative	Positive	Glycine
1	Licuraside	His41, Cys44, Thr190, Gln192	-	Val42, Cys44, Met49, Ala191, Met165, Ala193, Leu167, Pro168	Thr25, His41, Thr45, Ser46, Gln189, Thr190, Gln192, Thr169	-	Arg188	-
2	Glucoliquiritin apioside	Hie41, Glu166	-	Leu27, Val42, Cys44, Met49, Leu50, Cys145, Phe185, Val186, Met165, Leu167, Pro168, Ala191	Hie41, Thr25, Thr45, Ser46, Hie164, Gln189, Thr190, Gln192	Glu166, Asp187	Arg188	-
3	7,3′-Dihydroxy-5′-methoxyisoflavone	Gln192	-	Met49, Met165, Leu167, Pro168, Ala191	His41, Hie164, Thr169, Gln189, Thr190, Gln192	Glu166, Asp187	Arg188	-
4	Licuroside	Gln192	-	Ala193, Ala191, Pro168, Leu167, Met165, Met49, Tyr54, Phe185, Cys145	Gln192, Thr190, Gln189, His41, Hie164	Glu166, Asp187	Arg188	-
5	Reference	-	-	Pro122, Tyr118	Ser123, Ser121, Asn119	-	-	-

**Table 3:** Averaged binding free energies (kcal/mol) and energy dissociation components calculated using an MM/GBSA method for all the selected bioactive compounds and the reference compound docked with SARS-CoV-2 M^pro^.

Components	Energy (kcal/mol)				
	SARS-CoV-2 M^pro^-Licuraside	SARS-CoV-2 M^pro^-Glucoliquiritin	SARS-CoV-2 M^pro^-7,3′-Dihydroxy-5′-Methoxyisoflavone	SARS-CoV-2 M^pro^-Licuroside,	SARS-CoV-2 M^pro^-Glycyrrhizin
ΔG_Bind_	−58.66 ± 8.09	−80.0 ± 5.60	−42.73 ± 1.94	−42.93 ± 3.96	−23.42 ± 5.84
ΔG_Bind Coulomb_	−22.44 ± 4.91	−26.69 ± 5.40	−13.27 ± 2.54	−14.78 ± 2.82	43.97 ± 15.90
ΔG_Bind Covalent_	2.86 ± 1.93	3.58 ± 1.10	1.10 ± 1.13	2.08 ± 0.83	2.34 ± 2.26
ΔG_Bind Hbond_	−1.89± 0.39	−1.77 ± 0.59	−0.68 ± 0.18	−0.62 ± 0.21	−1.57 ± 1.13
ΔG_Bind Lipo_	−15.60 ± 2.20	−20.82 ± 0.67	−8.13 ± 0.28	−8.02 ± 0.47	−9.67 ± 1.52
ΔG_Bind Packing_	−1.48 ± 0.30	−1.62 ± 0.33	−0.91 ± 0.56	−1.7 ± 1.23	0 ± 0
ΔG_Bind SelfCont_	0 ± 0	0 ± 0	0 ± 0	0 ± 0	0 ± 0
ΔG_Bind Solv GB_	25.56 ± 3.48	33.06 ± 1.44	14.82 ± 1.69	17.06 ± 1.59	−35.14 ± 15.36
ΔG_Bind vdW_	−45.66 ± 4.54	−65.81 ± 2.69208	−35.64 ± 1.51	−36.94 ± 2.25	−23.35 ± 4.22
Lig Strain Energy	4.44 ± 1.59	5.77 ± 2.85	2.12 ± 0.80	2.07 ± 0.75	3.74 ± 2.04

## Data Availability

Data is contained within the article or Supplementary Material.

## Supplementary Materials

The following are available online at https://www.mdpi.com/article/10.3390/ph14121216/s1, Table S1: List of bioactive compounds from *Glycyrrhiza glabra* screened at the active pocket of SARS-CoV-2 M^pro^, Figure S1: 2D interaction poses for the docked poses of SARS-CoV-2 M^pro^ with reference compound, i.e., Glycyrrhizin. Herein, hydrogen bond formation (pink arrows), hydrophobic (green), polar (blue), red (negative), violet (positive), glycine (grey), and π-π stacking (green line), interactions are also depicted in the respective docked complexes.
