# Supplementary material for: Computational and In Vitro Experimental Investigations Reveal Anti-Viral Activity of Licorice and Glycyrrhizin against Severe Acute Respiratory Syndrome Coronavirus 2
Source: Pharmaceuticals (Basel). 2021 Nov 24;14(12):1216. doi: 10.3390/ph14121216 (PMC8703534; doi:10.3390/ph14121216)
Supplement: Supplementary file 1 [file pharmaceuticals-14-01216-s001.zip › pharmaceuticals-1448015 suppl.pdf]

Supplementary Information

Table S1. List of bioactive compounds from *Glycyrrhiza glabra* screened at the active pocket of SARS-CoV-2 M^{pro}.

S.no	Compound	PubChem ID	Docking score (kcal/mol)
1	Licuraside	14282455	-9.831
2	Glucoliquiritin-apioside	74819335	-9.558
3	7,3'-dihydroxy-5'-methoxyisoflavone	66728338	-9.423
4	Licuroside	6475724	-9.319
5	Kanzonol r	131753027	-9.037
6	Licochalcone a	5318998	-8.846
7	Neoisoliquiritin	5320092	-8.818
8	Formononetin	5280378	-8.754
9	Licoricone	5319013	-8.68
10	Isomucronulatol	602152	-8.679
11	Pubchem	5317764	-8.665
12	Licoisoflavone a	5281789	-8.649
13	Prunetin	5281804	-8.639
14	Licoisoflavone b	5481234	-8.561
15	Glyzaglabrin	5317777	-8.483
16	Isoliquiritin apioside	6442433	-8.475
17	Licoflavone b	11349817	-8.475
18	Licoflavone b	11349817	-8.475
19	Glabrene	480774	-8.463
20	Genistein	5280961	-8.323
21	Isoschaftoside	3084995	-8.238
22	Liquiritin	503737	-8.23
23	Licochalcone b	5318999	-8.145
24	Neoliquiritin	51666248	-8.115
25	Licocoumarone	503731	-8.104
26	5,7-dihydroxy-2-(4-hydroxyphenyl)chroman-4-one	932	-8.102
27	7-hydroxy-2-methylisoflavone	5380976	-8.042
28	Apigenin	5280443	-8.02
29	Glycyrrhisoflavanone	5317762	-8.011
30	Glabrone	5317652	-7.939
31	Glabridin	124052	-7.936
32	Liquiritigenin	114829	-7.864
33	Quercetin	5280343	-7.858
34	Glycycoumarin	5317756	-7.778
35	Echinatin	6442675	-7.701
36	Phaseollinisoflavan	162412	-7.698
37	Pinocembrin	68071	-7.678
38	Shinflavanone	197678	-7.665
39	6-c-beta-glucopyranosyl-8-c-alpha-arabinopyranosylapigenin	442658	-7.646
40	7-acetoxy-2-methylisoflavone	268208	-7.638
41	4',7-dihydroxyflavone	29503	-7.638
42	Licopyranocoumarin	122851	-7.628
43	Kumatakenin	5318869	-7.602
44	Glabranin	124049	-7.599
45	Isoliquiritigenin	638278	-7.581
46	Glabrol	11596309	-7.571
47	Liqcoumarin	11378967	-7.546
48	Isoliquiritin	5318591	-7.459
49	Licoflavonol	5481964	-7.415
50	Isoviolanthin	101422758	-7.4
51	2-(3,4-dimethoxy-phenyl)-3,5,6-trimethyl-pyrazine 1,4-dioxide	648543	-7.383
52	Hispaglabridin b	15228661	-7.382
53	Licoflavanone	14218027	-7.334
54	4-hydroxychalcone	5282361	-7.325
55	Galangin	5281616	-7.323
56	1-methoxyphaseollin	272877623	-7.314
57	Isoglycycoumarin	14187587	-7.314
58	5-o-methylglycyrol	5319666	-7.306
59	Astragalin	5282102	-7.247
60	Licoisoflavanone	392443	-7.245
61	7-methoxy-2-methylisoflavone	354368	-7.238
62	Hispaglabridin a	442774	-7.192
63	Thiamine	1130	-7.182
64	Isoquercitrin	5280804	-7.164
65	Isolicoflavonol	5318585	-7.061

66	Alpha-(phenyl-1,2,4-triazol-3-yl)-5-ethyl-2,4-dihydroxy-acetophenone oxime	137235235	-7.032
67	4-methylmercapto-2-methyl-5-isopropyl-phenol	121220586	-6.959
68	3-hydroxy-glabrol	480854	-6.929
69	Glyzarin	44257206	-6.923
70	Galactomannan-3d	439336	-6.906
71	Vitexin	5280441	-6.823
72	Isoglycyrol	124050	-6.816
73	1-furfuryl-2-acetyl-pyrrole	20560368	-6.801
74	3'-methoxyglabridin	5319439	-6.793
75	Shinpterocarpin	10336244	-6.789
76	Licoricidin	480865	-6.766
77	Estriol	5756	-6.75
78	Beta-sitosterol	222284	-6.692
79	Methoxsalen	4114	-6.648
80	Conformer3d	4114	-6.648
81	4'-o-methylglabridin	5319664	-6.602
82	2,4-difurfurylfuran	53423642	-6.6
83	9,12,13-trihydroxy-10,11-epoxyoctadecanoic acid	195302	-6.573
84	Euchrenone a16	636551	-6.565
85	Isoglabrolide	15559941	-6.551
86	Isoglabrolide	15559941	-6.551
87	Trifluoromethyl(2-(2-furyl)ethyl) ketone	102441980	-6.519
88	1-isopropyl-4-methyl-3-cyclohexen-1-ol	11230	-6.496
89	1-(2-furylmethyl)-1h-pyrrole-2-carbaldehyde	529880	-6.477
90	Thymol	6989	-6.386
91	Stigmasterol	5280794	-6.337
92	4-isopropylanisole	77783	-6.336
93	7-methoxycoumarin	10748	-6.304
94	Thujone	261491	-6.269
95	Paeonol	11092	-6.246
96	Uralsaponin b	163744	-6.204
97	Betulic acid	73759701	-6.115
98	N-acetylpyrrole	521947	-6.068
99	1-methoxy-4-propan-2-ylcyclohexane	23594959	-6.063
100	Umbelliferone	5281426	-6.059
101	2-hydroxy-4-methyl-benzaldehyde	61200	-5.993
102	Myrtenal	61130	-5.955
103	1-methylpyrrole-2-carboxaldehyde	14504	-5.954
104	Glycyrrhizin	3495	-5.92
105	Alpha-terpineol	17100	-5.875
106	Difurfuryl ether	263034	-5.851
107	Desoxoglabrolide	5257561	-5.836
108	Glycyrrhizic acid	14982	-5.815
109	Apioglycyrrhizin	195343	-5.776
110	2-(beta-d-glucopyranosyluronic acid)-d-glucuronic acid	440305	-5.768
111	Eugenol	3314	-5.762
112	4-propenyl-anisole	121216601	-5.736
113	2-acetyl-5-methylfuran	14514	-5.685
114	1,7-dimethyl-7(4-methyl-3-pentenyl)-tricycloheptane	12315252	-5.655
115	Sinapic acid	637775	-5.653
116	Fenchone	14525	-5.649
117	1-(2-furyl)-propan-2-one	228583	-5.622
118	D-glucuronic acid	94715	-5.617
119	Ethyl-linolenate	5367460	-5.592
120	2-[(e)-2-(furan-2-yl)ethenyl]furan	5373671	-5.553
121	Ferulic acid	445858	-5.541
122	5-pentyl-2h-pyran-2-one	71356827	-5.531
123	2,3,5,6-tetramethylpyrazine	14296	-5.512
124	Anethole	637563	-5.51
125	18alpha-hydroxyglycyrrhetic acid	14189465	-5.506
126	Glycyrrhetinic acid	3230	-5.449
127	4-hydroxybenzoic acid	135	-5.447
128	1-(5-methyl-2-furanyl)-1,2-propanedione	70968	-5.426
129	Ethyl-linoleate	5282184	-5.409
130	Guaiacol	460	-5.385
131	Araboglycyrrhizin	195342	-5.352
132	Tetramethyl pyrazine-2,3,5,6-tetracarboxylate	291621	-5.322
133	2-phenylethanol	6054	-5.309
134	Linalool	102611	-5.233

135	Estragole	8815	-5.152
136	Furfuryl-acetate	12170	-5.129
137	2-acetylpyrrole	14079	-5.107
138	Furfuryl propionate	61166	-5.085
139	1-methyl-2-pyrrolidinone	13387	-5.06
140	2-methyltetrahydrofuran-3-one	18522	-5.05
141	2,3-dihydrobenzofuran	10329	-5.043
142	2,6-dimethyl-pyrazine	7938	-5.037
143	Alnusenone	146159037	-5.003
144	2,2'-difurylmethane	70972	-4.946
145	2,2'-(1,2-ethylenediyl)bis(furan)	526614	-4.946
146	3-methyl-2(5h)-furanone-3d	30945	-4.907
147	2-pentylfuran	19602	-4.882
148	28-hydroxyglycyrrhetic acid	76064992	-4.849
149	7-methoxy-3,7-dimethyl-octanal	223668652	-4.846
150	Glabrolide	90479675	-4.718
151	2,3-dihydro-4-methyl-furan	36744	-4.699
152	Beta-amyrin	73145	-4.645
153	Furfuryl formate	556916	-4.62
154	3,24-dihydroxy-oleana-11,13(18)-dienoic-acid	2340547	-4.458
155	Liquoric acid	131751571	-4.345
156	11-deoxoglycyrrhetinic acid	12305517	-4.293
157	Glycyrrhetol	12310283	-4.23
158	Lupeol	259846	-4.219
159	3-methyl-3-hepten-2-one	5364798	-3.971
160	Liquiritic acid	112111	-3.946
161	Liquiritic acid	112111	-3.946
162	18 beta-glycyrrhetinic acid	44435791	-3.946
163	Beta-glycyrrhetinic-acid	44435791	-3.946
164	Glabric-acid	46173993	-3.879
165	18-alpha-glycyrrhizinic-acid	158471	-3.865
166	Enoxolone	10114	-3.765
167	Licoricesaponin g2	14891565	-3.679
168	Glyyunnansapogenin b	131752021	-3.654
169	Betulinic acid	64971	-3.617
170	Soyasaponin ii	443614	-3.485
171	Glycyrrhetic acid	5343385	-3.143
172	Heptane-1,2-diol	77302	-2.955
173	Glycyrram	53393640	-2.71

Results and Discussion

Virtual screening and re-docking analysis

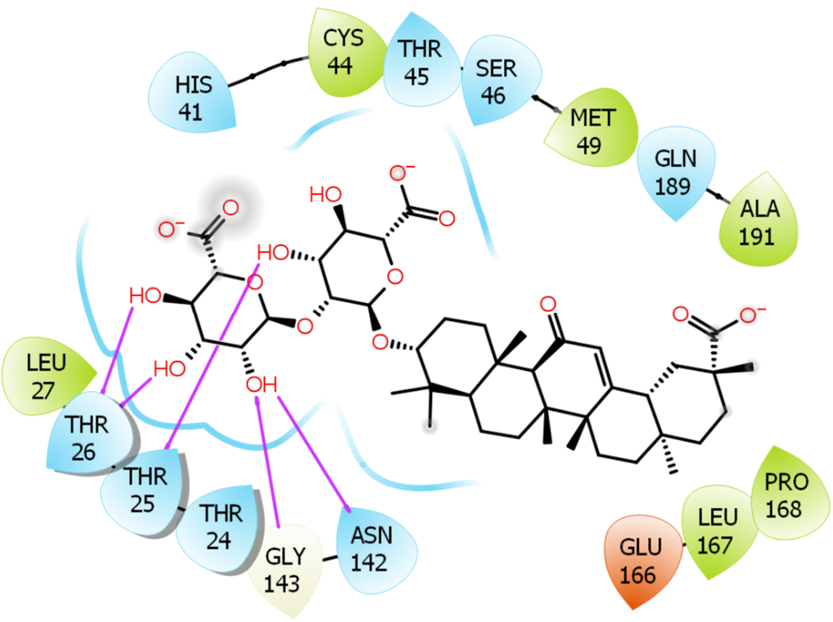

Figure S1. 2D interaction poses for the docked poses of SARS-CoV-2 M^{pro} with reference compound, i.e. Glycyrrhizin. Herein, hydrogen bond formation (pink arrows), hydrophobic (green), polar (blue), red (negative), violet (positive), glycine (grey), and π-π stacking (green line), interactions are also depicted in the respective docked complexes.
